# Morphological and Molecular Characterization of *Punctodera Stonei* Brzeski, 1998 (Nematoda: Heteroderidae) from Virginia, USA

**DOI:** 10.2478/jofnem-2022-0008

**Published:** 2022-04-16

**Authors:** Mihail R. Kantor, Sergei A. Subbotin, Gregory Huse, Zafar A. Handoo

**Affiliations:** 1 Mycology and Nematology Genetic Diversity and Biology Laboratory, USDA, ARS, Northeast Area, Beltsville, MD 20705, USA; 2 California Department of Food and Agriculture, Plant Pest Diagnostic Center, 3294 Meadowview Road, Sacramento, CA 95832, USA; 3 Center of Parasitology of A.N. Severtsov Institute of Ecology and Evolution of the Russian Academy of Sciences, Leninskii Prospect 33, Moscow 117071, Russia; 4 Arlington National Cemetery, 1 Memorial Avenue Arlington, VA 22211, USA

**Keywords:** cyst nematode, grass, *Punctodera stonei*, Virginia, United States, detection, diagnosis, systematics, taxonomy

## Abstract

In August of 2021, several cysts with juveniles and eggs were discovered during a vegetation survey conducted at the Arlington National Cemetery, Virginia. Eight soil samples were collected from the rhizosphere region of the common grass (*Festuca arundinacea L.*) and processed at the Mycology and Nematology Genetic Diversity and Biology Laboratory (MNGDBL). Cysts were light to dark brown in color, and oval to pear-shaped without bullae in young cysts but present in older cysts and with prominent vulval cone. The juveniles had slightly concave stylet knobs projecting sometimes anteriorly, tail tapering gradually to a narrowly rounded terminus, and hyaline tail terminus conspicuous at least twice the length of stylet. The molecular analysis included the analysis of three gene sequence fragments: D2–D3 of 28S rRNA, ITS rRNA, and COI. The nematode species was identified by both morphological and molecular means as Stone's cyst nematode, *Punctodera stonei*. Detection of *P. stonei* in Virginia represents a new record of this species in the United States, and a second report after Canada in North America.

*Punctodera stonei* (Brzeski, 1998) is a species belonging to the genus *Punctodera* (Mulvey and Stone, 1976). The genus also includes other species, namely: *P. punctata* (Thorne, 1928; Mulvey and Stone, 1976), *P. matadorensis* (Mulvey and Stone, 1976), *P. chalcoensis* (Stone *et al*., 1976), and two species *P. mulveyi* (Kantor *et al*., 2020) and *P. achalensis* (Lax et al., 2021) described in the last 2 years. Until today, only *P. punctata* was reported from several states in the USA (Subbotin *et al*., 2010).

Stone's cyst nematode, *P. stonei*, was first described from grass lawn collected in Skirniewice, Poland by Brzeski (1998). *P. stonei* has been found in Europe, where it was reported from several countries (Subbotin *et al.*, 2010), and also in Canada (Kantor *et al*., 2020).

In August 2021, several cysts were recovered from soil samples collected in and around common grass (*Festuca arundinacea* L.) during a natural vegetation survey conducted at the Arlington National Cemetery. The soil samples were processed at the Mycology and Nematology Genetic Diversity and Biology Laboratory (MNGDBL), where the morphological characteristics of cysts and juveniles were analyzed and recorded. The molecular identification was conducted at the California Department of Food and Agriculture.

Based on the results of morphological and molecular studies, this nematode is identified herein as *P. stonei* (Brzeski, 1998). The goal of this study is to provide a brief morphological and molecular characterization of this *P. stonei* population from Virginia, United States.

## Materials and Methods

### Morphological study

**Figure 1:**
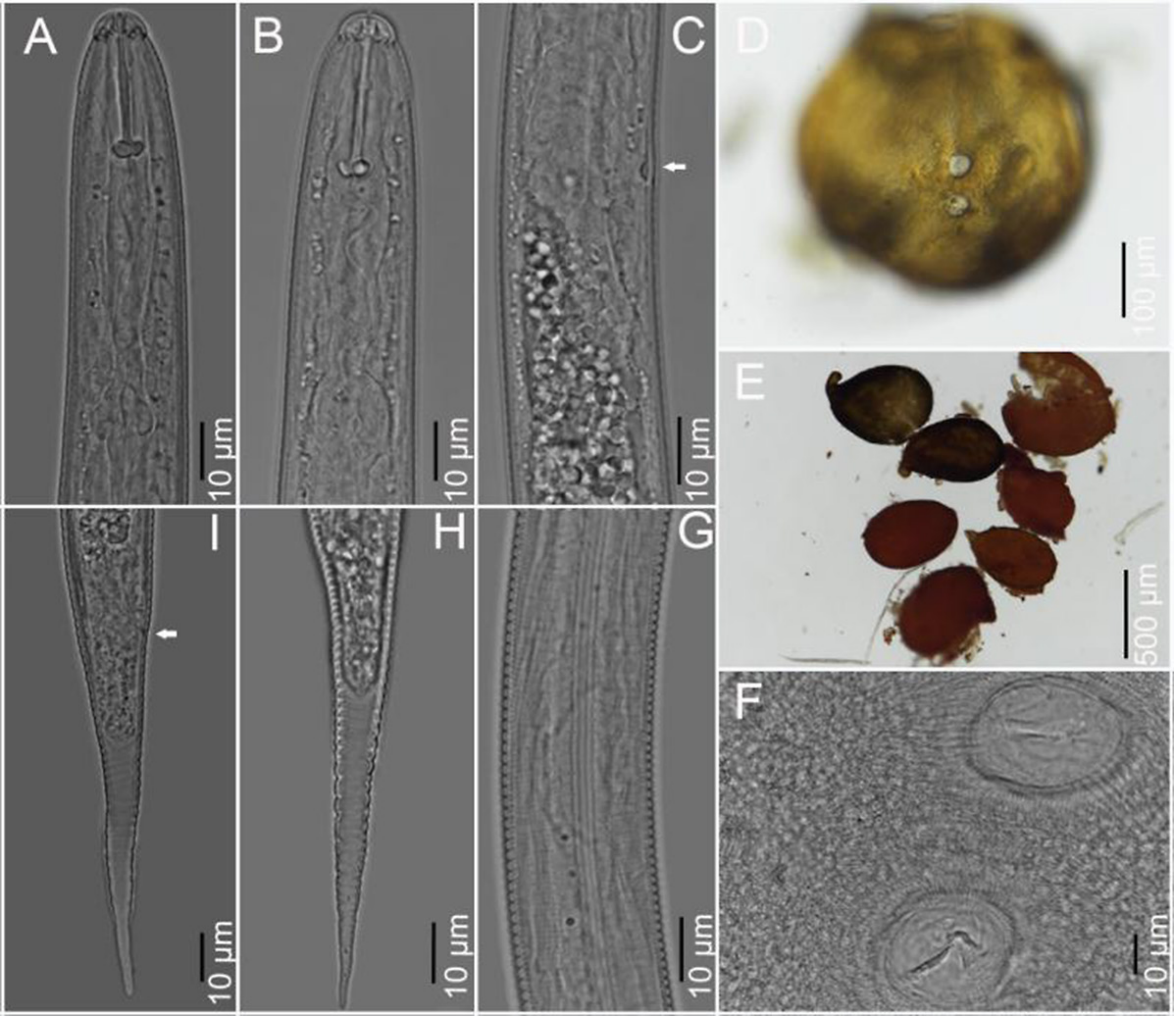
Photomicrographs of cysts, vulval cones, and J2 of *P. stonei* from Virginia. A, B: Anterior ends of J2s; C: Excretory pore and hemizonid with arrow pointing toward hemizonid; D, E: Entire cysts with D showing both fenestra in the middle; F: Cyst posterior part showing vulval and anal fenestrae; G: Lateral field with four incisures for J2; H, I: Tails of J2s with arrow pointing toward the anal area in I.J2, second-stage juveniles.

Cysts, second-stage juveniles (J2), and eggs were obtained from eight Virginia soil samples collected from around tall fescue (*F. arundinacea* L.), from a location with the GPS coordinates: 38°52′28.4″ N, 77°03′49.8″W. Juveniles were fixed in 3% formaldehyde and processed to glycerin by the formalin glycerin method (Hooper, 1970; Golden, 1990). Cysts contained viable eggs and J2, which were examined morphologically and molecularly for species identification. Observations of morphological characters critical for identification were cyst shape, color and nature of fenestration, cyst wall pattern, J2 stylet length, shape of stylet knobs, and shape and length of tail and hyaline tail terminus (Fig. 1). Photomicrographs of cyst vulval cones and J2 were made with an automatic Nikon Eclipse Ni compound microscope (Nikon Instruments, NY) using a Nikon DS-Ri2 camera (Nikon Instruments, NY). Measurements were made with an ocular micrometer on a Leitz DMRB compound microscope (Leica Microsystems, IL). All measurements are in micrometers.

### DNA extraction, PCR, and sequencing

DNA was extracted from several juveniles using the proteinase K protocol. DNA extraction and PCR protocols were used as described by Subbotin (2021a). The following primer sets were used for PCR: (i) forward D2A (5′ – ACA AGT ACC GTG AGG GAA AGT TG – 3′) and reverse D3B (5′ – TCG GAA GGA ACC AGC TAC TA – 3′) primers for amplification of the D2–D3 expansion region of the 28S rRNA gene; (ii) forward TW81 (5′ – GTT TCC GTA GGT GAA CCT GC – 3′) and reverse AB28 (5′ – ATA TGC TTA AGT TCA GCG GGT – 3′) primers for amplification of the ITS1-5.8-ITS2 rRNA gene; (iii) forward Het-coxiF (5′ – TAG TTG ATC GTA ATT TTA ATG G – 3′), and reverse Het-coxiR (5′ – CCT AAA ACA TAA TGA AAA TGW GC – 3′) primers for amplification of the partial *COI* gene of mtDNA (Subbotin *et al*., 2021a). Sequencing was conducted at the Genewiz (CA, USA). The newly obtained sequences were submitted to the GenBank database under accession numbers given on phylogenetic trees.

### Phylogenetic and sequence analysis

The new sequences for the rRNA and mtDNA genes were aligned using ClustalX 1.83 (Chenna *et al*., 2003) with their corresponding published gene sequences of nematodes from the genus *Punctodera* (Sabo *et al*., 2002; Subbotin *et al*., 2006; Toumi, *et al*., 2013; Skantar *et al*., 2019; Kantor *et al*., 2020). Sequence alignments were analyzed with Bayesian inference (BI) using MrBayes 3.1.2 (Ronquist and Huelsenbeck, 2003). BI analysis for each gene was initiated with a random starting tree and was run with four chains for 1.0 × 10^6^ generations as described by Subbotin (2021b).

## Results and Discussion

### Measurements and description

Measurements of J2 from Virginia (*n* = 10) included lengths of body = 557.3 ± 30.0 (505.0–595.0) μm, stylet well developed = 26.8 ± 0.7 (26.0–27.5) μm, with slightly concave basal knobs, tail length = 80.6 ± 3.7 (75.0–86.0) μm, and hyaline tail terminus = 50.6 ± 3.4 (45.0–55.0) μm. The lateral field had four narrow, distinct lines. Tail length/hyaline region ratio = 1.6 ± 0.1 (1.5–1.7), a = 23.3 ± 1.4 (20.2–25.2), b = 3.1 ± 0.3 (2.7–3.6), c= 6.9 ± 0.3 (6.5–7.4), and c′ = 6.1 ± 0.5 (5.2–6.5). Measurement of J2 from several populations given in Subbotin *et al*. (2010) include body length 520.0 (470.0–590.0) μm; a = 26.0 (23.0–29.0); c = 6.7 (5.9–7.9); c′ = 5.7 (4.5–6.7); stylet length = 25.4 (24.0–26.5) μm; tail length = 78.0 (60–95) μm; and hyaline tail terminus = 50.0 (41.0–66.0) μm. Shapes of the tail, tail terminus, and stylet knobs were consistent with those of *P. stonei* (Brzeski, 1998). The cysts (*n* = 5) were light brown to dark brown in color, and oval to pear-shaped without bullae in younger cysts but present in older cysts and with prominent vulval cone, with two fenestrae almost identical in size. Cyst wall with a wavy to ridge-like or sometimes zigzag pattern in the middle and with abundant punctations (Fig. 1F) was observed. Fenestra length (*n* = 6) = 30.5 ± 4.0 (25.1–35.0) μm; fenestra width = 29.5 ± 5.0 (25.0–38.0) μm; and the distance between the two fenestrae = 28.7 ± 3.9 (25.0–30.4) μm. Cyst length/width ratio = 1.4 ± 0.4 (0.8–1.7) μm. Vulval slit length (*n* = 2) = 8.0 μm, 12.5 μm. The fenestra width, length, and vulval slit to anus distance of the Virginia population is within the range reported for the type population. No males were found. The morphometrics and morphology of the cysts were also consistent with those of the type population of *P. stonei* (Brzeski, 1998).

### Molecular characterization

#### The D2–D3 expansion region of 28S rRNA gene

**Figure 2:**
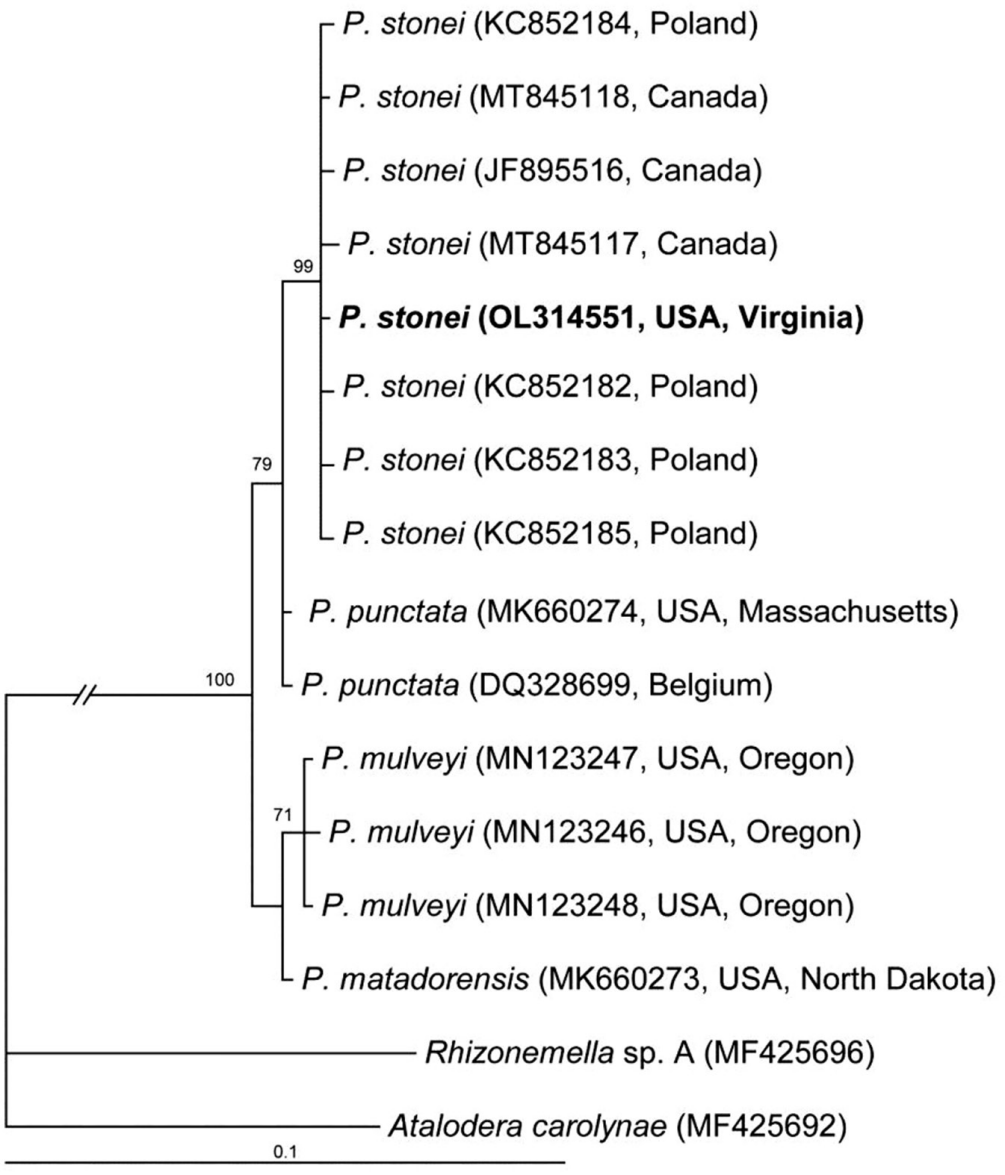
Phylogenetic relationships within the genus *Punctodera*: Bayesian 50% majority rule consensus tree from two runs, as inferred from analysis of the D2–D3 of 28S rRNA gene sequence alignment under the GTR + I + G model. Posterior probabilities and bootstrap values ≥70% are given for appropriate clades. New sequences are indicated by bold font.

The alignment contained 14 sequences of the *Punctodera* species and two sequences of the outgroup taxa, and it had 757 bp in length. Phylogenetic relationships within the genus are given in Figure 2. The D2–D3 of 28S rRNA gene sequence of *P. stonei* from Virginia formed a clade (posterior probability, PP = 99%) with that of this species from Poland and Canada. The interspecific sequence variation within *P. stonei* was up to 0.4% (3 bp).

#### The ITS rRNA gene

**Figure 3:**
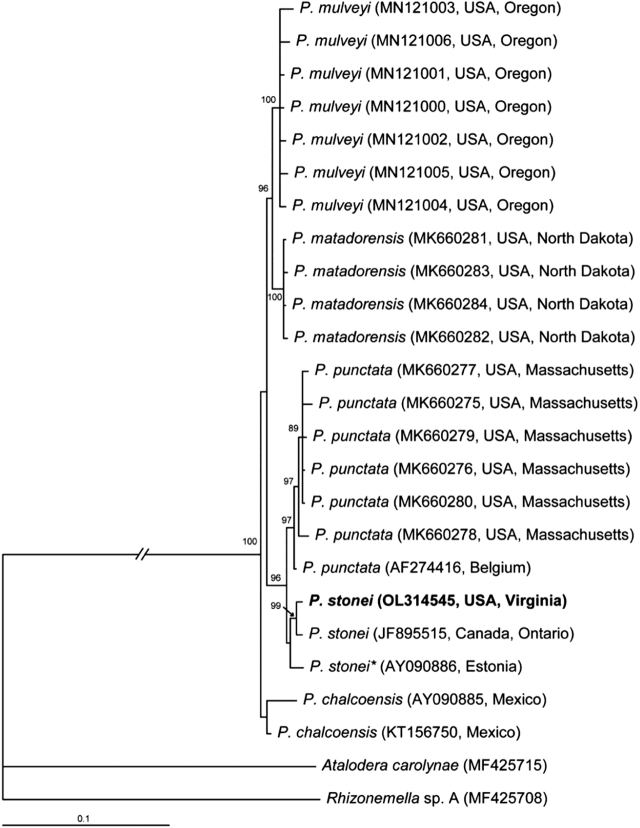
Phylogenetic relationships within the genus *Punctodera*: Bayesian 50% majority rule consensus tree from two runs, as inferred from analysis of the ITS rRNA gene sequence alignment under the GTR + I + G model. Posterior probabilities and bootstrap values ≥70% are given for appropriate clades. New sequences are indicated by bold font. *Identified as *P. punctata* in the GenBank and by Sabo *et al*. (2002).

The alignment contained 23 sequences of the *Punctodera* species and two sequences of the outgroup taxa, and it had 990 bp in length. Phylogenetic relationships within the genus are given in Figure 3. The ITS rRNA gene sequence of *P. stonei* from Virginia formed a clade (PP = 99%) with that of this species from Canada. The interspecific sequence variation within *P. stonei* was up to 1.1% (10 bp).

#### The COI gene

**Figure 4:**
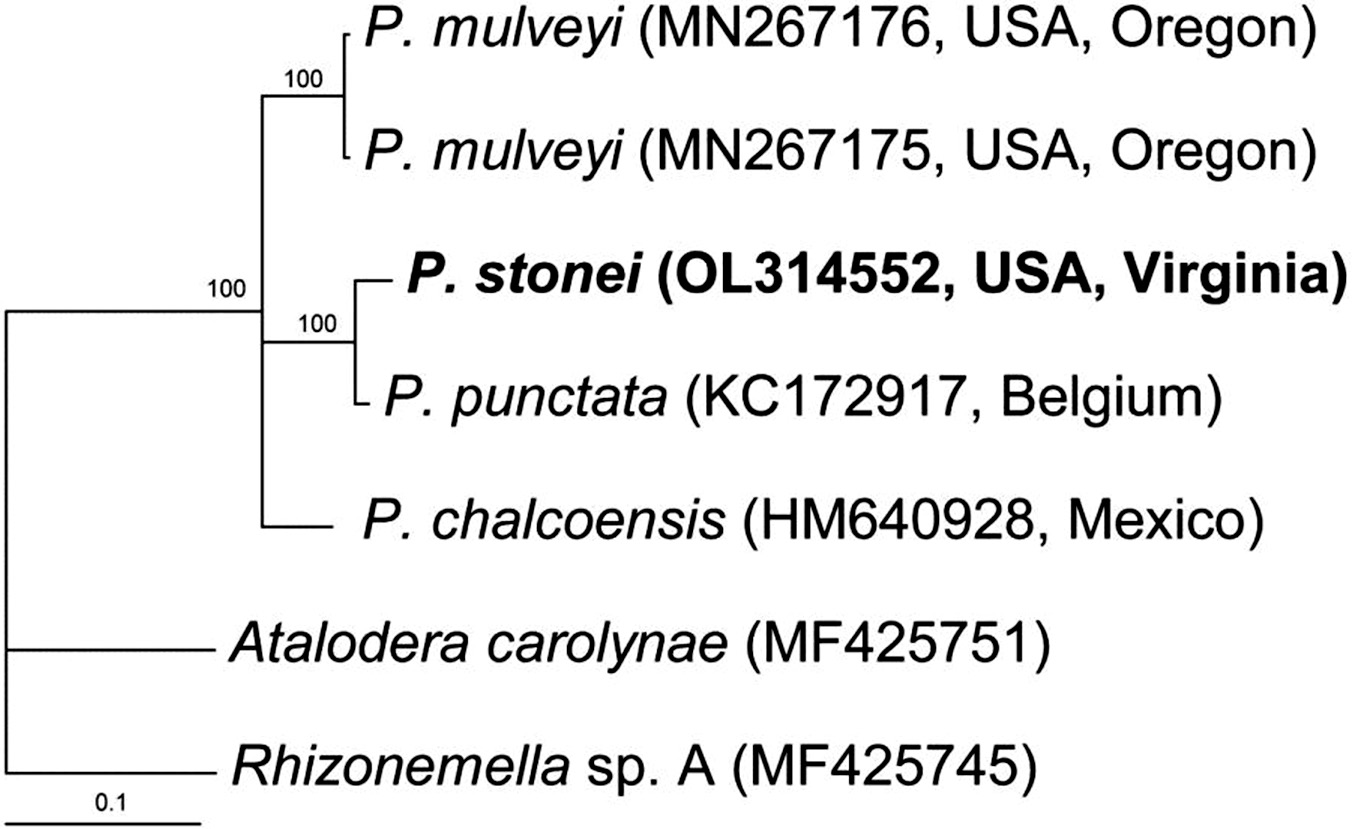
Phylogenetic relationships within the genus *Punctodera*: Bayesian 50% majority rule consensus tree from two runs, as inferred from analysis of the *COI* gene sequence alignment under the GTR + I + G model. Posterior probabilities and bootstrap values ≥70% are given for appropriate clades. New sequences are indicated by bold font.

The alignment contained five sequences of the *Punctodera* species and two sequences of the outgroup taxa, and it had 990 bp in length. Sequence of *P. stonei* differed from that of *P. punctata* in 2.0% (8 bp). Phylogenetic relationships within the genus are given in Figure 4.

Based upon these collective morphological and molecular data, we identify this nematode as *P. stonei.* To our knowledge, this is the first report of the *P. stonei* from Virginia, USA, and a second report after Canada in North America.
